# Polymorphism of Interleukin-1 Gene Cluster in Polish Patients with Acute Coronary Syndrome

**DOI:** 10.3390/jcm10050990

**Published:** 2021-03-02

**Authors:** Tomasz Rechciński, Bożena Szymańska, Karina Wierzbowska-Drabik, Magdalena Chmiela, Agnieszka Matusiak, Małgorzata Kurpesa, Janusz Wróblewski, Jarosław D. Kasprzak

**Affiliations:** 1 Department and Chair of Cardiology, Medical University of Lodz, 91-347 Lodz, Poland; karina.wierzbowska-drabik@umed.lodz.pl (K.W.-D.); malgorzata.kurpesa@umed.lodz.pl (M.K.); jaroslaw.kasprzak@umed.lodz.pl (J.D.K.); 2 Core-Lab, Medical University of Lodz, 90-236 Lodz, Poland; bozena.szymanska@umed.lodz.pl; 3 Department of Immunology and Infectious Biology, Faculty of Biology and Environmental Protection, University of Lodz, 90-237 Lodz, Poland; magdalena.chmiela@biol.uni.lodz.pl (M.C.); agaow@wp.pl (A.M.); 4 Department of Translation Studies, Institute of English Studies, University of Lodz, 90-236 Lodz, Poland; jzwrob@gmail.com

**Keywords:** interleukin-1, gene polymorphism, acute coronary syndrome, atherosclerosis

## Abstract

Background and objectives: Some experimental studies demonstrated adverse modulation of atherothrombosis by interleukin-1beta (IL-1b). To assess the relationship between the five most common variants of three polymorphisms of the IL1b gene cluster and the complexity of coronary atherosclerosis expressed in Gensini Score (GS), and the age of onset of the first acute coronary syndrome (ACS), we assessed the patients (pts) hospitalized due to ACS in this aspect. Materials and Methods: 250 individuals were included. The single nucleotide polymorphisms of IL1b gene: transition T/C at -31 position, C/T at -511, and those of IL1 receptor antagonist gene (IL1RN)—variable number of tandem repeats allele 1, 2, 3, or 4—were determined by PCR. GS was calculated from the coronary angiogram performed at the index ACS. The impact of the presence of T or C and allele 1 to 4 at the investigated loci on the mean GS, GS greater than 40, mean age of onset of ACS, and the fraction of pts over 60 years of age at ACS were compared between the five most common genotype variants. Results: The five most common variants were present in 203 pts (81.2%). Patients with pair 22 in ILRN had the lowest rate and those with pair 12 had the highest rate of ACS before 60 years of age (29.4 vs. 67.8%; *p* = 0.004). GS > 40 entailed an eight-fold increase of risk, as observed when pts with one T allele at locus -31 were compared with carriers of 2 or no T allele at this locus: OR 8.73 [CI95 4.26–70.99] *p* = 0.04. Conclusion: Interleukin-1 beta is subject to frequent genetic variability and our results show a potential relationship of this polymorphism with the extent of coronary atherosclerosis and age at the first ACS.

## 1. Introduction 

Several experiments have demonstrated adverse modulation of atherogenesis and thrombosis by interleukin-1beta (IL-1b). Starting from the selectin-dependent rolling of leukocytes on the surface of the vascular endothelium, through their integrin-mediated adhesion and activation, to the transmigration of monocytes to the arterial wall and the formation of activated macrophages—at every stage, IL-1b increases the dynamics of these phenomena [1,2]. Additionally, the processes connected with the destabilization of the atherosclerotic plaque—such as collagen degradation in the outer layer of the plaque or necrosis of the lipid core as a consequence of hypoxia and pyroptosis—are the adverse effects of IL-1b on the cells in the atherosclerotic plaque or those migrating to it [3,4]. Platelets, vital for the pathogenesis of acute coronary syndromes (ACS), are both the objective of IL-1b and its abundant source. Those prokaryotic morphotic elements of blood, with their markers reflecting the level of inflammatory stimulation and cardiac risk, contain the large amounts of mRNA needed for the synthesis of IL-1b [5,6]. Clinical data confirm the contribution of imbalance between the activity of IL-1b and the concentration of its natural receptor antagonist—IL-1RA to the pathogenesis of inflammatory diseases [7]. Atherosclerosis is a process which involves a milder degree of imbalance of the IL-1b/IL-1RA system than that observed in chronic inflammatory diseases such as rheumatoid arthritis, psoriasis, gout, or inflammatory bowel disease [8,9,10].

In 2011, a multi-center CANTOS study was initiated to treat myocardial infarction (MI) survivors with monoclonal antibody against IL-1b (canakinumab). The hypothesis assuming that this treatment would reduce the risk of acute coronary syndromes (ACS) in patients with increased hsCRP > 2 mg/L was confirmed, and the results of the study were published in 2017 [11].

Genetic tests give unchanged result through the entire human life. Benefits of a strict anti-inflammatory treatment (canakinumab or colchicine) have been proven so far for myocardial infarction survivors in secondary prevention of cardiovascular events [12,13]. This study may be regarded as a part of research on markers for individual traits for intense inflammatory reactions which facilitate the progression of atherosclerosis during its asymptomatic period in people with classical risk factors and which may be helpful in identifying young individuals at risk.

The intensity of the biosynthesis of IL-1b and IL-1RA is determined by gene polymorphism. We selected *IL-1B* single nucleotide polymorphisms (SNPs) of the promotor region (responsible for the intensity of biosynthesis) located at loci -31 and -511 to evaluate its significance for an individual course of ischemic heart disease (IHD). For the locus -31, the more frequent (wild) allele is the thymine nucleotide (T) and the less frequent (mutated)—the cytosine (C). For the locus -511, the wild allele is C, and the mutated one—T. Other polymorphisms (not examined here) are also known (e.g., at loci +3954 or −1470).

The next polymorphism to be evaluated in this study concerns the IL-1RA gene (*IL-1RN*). It depends on the variable number of tandem repeats (VNTR) of a DNA sequence composed of 86 base pairs (bp), found in intron 2. The nomenclature of this polymorphism is presented in Table 1. *IL-1RN* VNTR polymorphism has been correlated with many diseases (recurrent miscarriages, carcinomas, etc.). 

We decided to test the hypothesis that the *IL-1B* genetic variants consisting of alternations between nucleotides containing C and T at loci: -31 and -511, and the variants of *IL-1RN* VNTR polymorphism can be connected with a different clinical course of IHD in ACS patients. Thus far, there have been no studies on the significance of the IL-1 gene cluster polymorphisms in Polish ACS patients. The specific aims were to evaluate the distribution of variants of *IL-1B* genes (at loci -31 and -511) and IL-1RA in patients after ACS, compare them with results of other studies, and try to determine the characteristics of patients (at their first ACS) in terms of the most frequent *IL-1B* and *IL-1RN* genetic variants, including the age at which they had their first ACS and the degree of coronary atherosclerosis assessed during coronary angiography performed during that episode.

## 2. Materials and Methods

In total, 250 Caucasian patients of the Department of Cardiology between 2006 and 2012, treated for ACS with or without ST-segment elevation, with the atherosclerotic substrate of myocardial ischemia confirmed by coronary angiography consistent with diagnosis of MI type 1, were included in the study [14]. The inclusion criteria were:

- >18 years of age,
- Having signed an informed consent to participate in the study including genetic tests.

We excluded patients in whom the ACS was caused by:

- An isolated critical change in the main left coronary artery,
- Coronary artery spasm and coronary embolism,
- Endocrine disorders or other conditions contributing to the imbalance between oxygen supply and demand in the myocardium (type 2) or necrosis of the myocardium associated with treatment procedures (type 4a and 4b).

The diagnosis of MI and treatment (both interventional and pharmacological) were in accordance with the guidelines for the diagnosis and treatment of ACS taking effect during enrollment of patients (published in 2007, 2008, and 2012) [15,16,17].

### 2.1. Group Characteristics

The demographic and clinical data of the participants are presented in Table 2, Table 3 and Table 4. 

**Figure 1:**
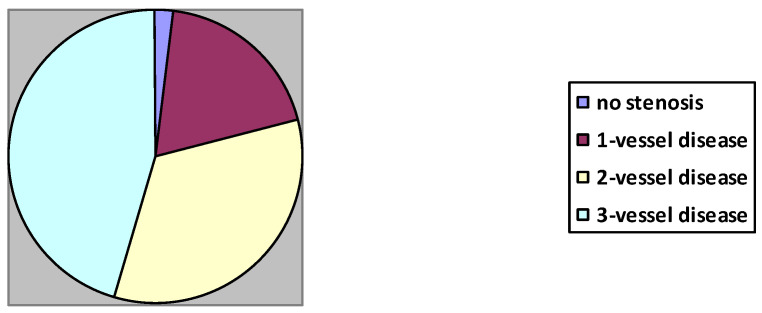
Distribution of coronary diffuseness in the investigated group: 114 patients had 3-vessel disease, 83—2-vessel disease, 48—1-vessel disease and 5 had no significant stenosis in the coronary arteries.

Angiographic characteristics are depicted in Figure 1. 

### 2.2. Study Protocol

Venous blood to determine genetic variants was sampled after the patients signed an informed consent. Having received genetic tests results, we identified demographic and angiographic profiles of patients with an identical genetic variant of *IL-1B* at loci-511 and -31 and of *IL-1RN* VNTR polymorphism. Groups homogenous in terms of genetic variants were compared according to the age of onset of first ACS and the degree of progression of coronary atherosclerosis using methods of statistical inference.

### 2.3. Genotyping

The DNA was isolated from 200 μL of blood (taken to EDTA and kept in −80 °C) using the QIAamp DNA Blood Mini Kit (Qiagen, Hilden, Germany) according to the producer’s instructions. SNPs in the *IL-1B* promotor sequence were examined by means of real-time PCR using TaqMan® SNP Genotyping Assay (Applied Biosystems, Foster City, CA, USA): for the polymorphism at locus-31 T > C (rs 1143627) Assay ID C_1839944_10, and at locus-511 C > T (rs 16944) Assay ID C_1839943_10. 

For the VNTR polymorphism of the *IL-1RN* gene, we used PCR with the starters IL1RNfor:5′-CTCAGCAACACTCCTAT-3′ and IL1RNrev:5′-TCCTGGTCTGCAGGTAA-3′. 

The products of the PCR were analyzed in 1% agarose gel or on microchips using the Microchip Electrophoresis System MultiNA (Shimadzu Corp., Kyoto, Japan).

Coronary catheterization was performed using INNOVA 2000 angiography system (General Electrics, Boston, MA, USA). Examinations were performed with radial access, and in the case of difficulties—with femoral access. The degree of atherosclerotic stenosis in the coronary artery responsible for the ACS and in the other arteries was evaluated first visually and then by means of a qualitative comparative analysis of the vessel diameter before the stenosis and at maximum stenosis. The right coronary artery was evaluated in at least three projections, and the left artery in five. For each patient, we recorded the presence or absence of collaterals and calculated the Gensini Score (GS)—the sum of products of values dependent on the degree of stenosis and the location of the plaque [18]. The stenosis >70% of coronary artery diameter was considered diagnostic for significant disease (for definition of 1-, 2-, and 3-vessel coronary disease) and the stenosis >50% of the left main coronary artery was considered equivalent to 2-vessel disease. The collaterals were defined as described by Rentrop et al., and grades 1–4 during balloon inflation were adjudicated as the presence of collaterals [19].

Two-dimensional M-mode echocardiograms (Vivid E9, GE) of all participants were obtained by a trained cardiologist. Left ventricular (LV) dimensions were obtained in parasternal long axis view, with measurement of interventricular septal thickness (IVST), left ventricle internal dimension in diastole (LVIDd), LV internal dimension in systole (LVIDs), and LV posterior wall thickness (PWT) according to the guidelines of the American Society of Echocardiography. Left ventricle mass (LVM) was measured according to the Penn convention using the Devereux formula. LVH was considered present if the LVM index (LVMI) was ≥115 g/m^2^ and 95 g/m^2^ for males and females, respectively. 

### 2.4. Statistical Methods

The distribution and dispersion of numeric variables were calculated; for variables with normal distribution, those were the arithmetic mean ± SD, and for variables whose distribution differed significantly from the normal—the median and interquartile range. We also gave minimum and maximum values. To assess the conformity of the distribution of variables with normal distribution, the Shapiro-Wilk test was used. Qualitative characteristics of the participants are presented in the form of frequency distribution and prevalence. 

The distribution of alleles and of genotypes was analyzed using the chi^2^ test. The analysis of the correlation of the GS and the genotype was done using the Kruskal-Wallis test. Rejecting the least frequent genetic variants (<0.5%) for the GS, we also performed an analysis of variance. Correlation between the genetic variant and the GS tertiles, the age at which the first ACS occurred, and the presence of collaterals was analyzed using the chi^2^ test and Fisher’s exact test, and in the case of the GS, the quantitative scales were also analyzed using linear regression. The statistical analyses were performed using the PQStat statistical packet, Version 1.4.4.126. *p* < 0.05 was regarded as significant and *p* < 0.01 as highly significant. Correlations at *p* < 0.1 but *p* ≥ 0.05 was regarded as a trend.

All authors declare that they have no conflict of interest. All procedures performed in the studies involving human participants were in accordance with the ethical standards of the institutional and national research committees and with the ethical guidelines of the 1975 Declaration of Helsinki. The local Bioethics Committee approved the study—resolution RNN/187/2001/KE and RNN/23/09/KE. Informed consent was obtained from all individual participants included in the study. Inclusion in the study did not affect further treatment. 

## 3. Results

The distribution of the alleles and genotypes.

The prevalence of specific alleles in the three studied loci was:

- For locus -31 of the *IL-1B* gene: T—66%, C—34%,
- For locus -511: C—65.8%, T—34.2%,
- For the *IL-1RN* gene—allele 1: 77.4%; 2: 18.6%; 3: 3.8%, and 4: 0.2%.

Data on the frequencies of the specific alleles, both the real and expected distribution of genotypes and the statistical analysis of the conformity of the real distribution with the expected distribution, are presented in Table 5.

No significant differences were observed between the real and expected frequencies of genotypes at loci -31 and -511 (*p* = 0.38 and 0.34, respectively). However, the chi^2^ test showed one significant difference between the real and expected distribution of the *IL-1RN* genotypes, i.e., homozygotes 11, 22, and 33 occurred more frequently than predicted by the Hardy-Weinberg equation, while heterozygotes 12, 13, and 14 occurred less frequently. 

A similar analysis was done for a combination of three polymorphisms (-31, -511, and IL-1 receptor antagonist). TT homozygotes at locus -31, CC at -511 and 11 in the *IL-1RN* gene and CT heterozygotes at locus -31, and CT at locus -511 and 11 or 12 in the *IL-1RN* were observed 2–3 times more frequently (*p* < 0.001) than predicted by the Hardy-Weinberg equation—in Table 5, these are variants number v, iv, and i. There were only 21 variants from among the 90 combinations theoretically possible at those three loci (nine variants for loci -31/-511 × 10 variants in the *IL-1RN* gene).

Variants CC/CC, TT/TT at loci -31 and -511 were absent in our group, and in the case of the *IL-1RN* gene, not a single individual carried the variant with pairs 23, 24, 34, and 44 at this locus. The absence of such combinations eliminates 48 variants from the pool of potential genotypes (circa 29% in the theoretical distribution). Some variants with very low expected frequency (<1:100) were overexpressed—five such variants accounted for 10.4% of the studied group; variants xii–xiv. Twelve patients (4.8%) carried variants whose theoretical frequency was <1:1000 (xvii–xxi in Table 6). For the purposes of clarity—groups with the five most numerous variants were assigned letter symbols. Patients carrying the five most frequent variants—XL, L, M, S, and XS—constituted 81.2% of the study group—Table 6. 

Polymorphisms at loci -31, -511, and -RN and the age of the first MI.

The mean age (±SD) at the first MI in our group was 59 ± 9, and the median was 60 years. We adopted 60 years as the threshold of the division between the MI at a younger or older age. In all the categories according to the genotype at locus -31 or -511, the differences in the distribution of patients who had their MI before 60 and after 60 were nonsignificant—Table 7 and Table 8. 

However, dividing patients according to the VNTR polymorphism of the *IL-1RN* made it possible to distinguish a group of 17 carriers of two alleles 2 (22), among whom patients with MI before 60 were only 29.4%, and carriers of two rare alleles 3 (33), among whom 40% were patients with MI at a younger age. The highest percentage of patients with MI at a younger age—67.8% out of 159 people—was among variant 12. Among the homozygous carriers of the most frequent allele 1 (the pair 11), the percentage of MI survivors before 60 was 48.4. The significance of the differences for the *IL-1RN* VNTR polymorphism, checked using the chi^2^ test and Fisher’s exact test, was *p* = 0.04 and *p* = 0.02, respectively—Table 9. 

Some known confounders of the age of onset of the first ACS are arterial hypertension, diabetes mellitus, and cigarette smoking—the distribution of these risk factors, verified by the chi^2^ test, was not different from random in the XL, L, M, S, and XS subgroups: *p* = 0.16; 0.65 and 0.61, respectively. 

**Figure 2:**
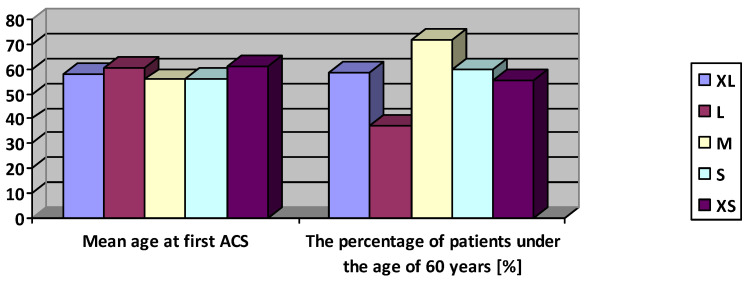
The comparison of mean age and the percentage of patients at the age under 60 years during first acute coronary syndrome depending on the combination of genetic variants.

In the combined analysis of two polymorphisms, -511 and -RN, there was still a significant difference (*p* = 0.04) in the distribution of the younger vs. older age of the first MI. It was connected with patients with MI at a younger age, carrying the following variants: -511CT/-RN11—27 out of 72 people (37.5%); -511CT/-RN12—29 out of 40 (72.5%), and -511TT/-RN22—2 out of 7 people (28.7%). In analogous analysis of two polymorphisms, -31 and -RN, the difference of distribution showed a trend resulting from 37% of patients with MI before 60 in the group -31CT/-RN11 and 72% in the group -31CT/-RN12 (*p* = 0.05 in Fisher’s exact test). Comparing the mean age of the first ACS did not show any significant difference of this parameter between the subgroups (*p* = 0.28)—Figure 2. The logistic regression analysis also failed to demonstrate a connection between the number of T alleles at loci -31 and -511 and the risk of MI before 60 (for locus -31 *p* = 0.86, and for locus -511 *p* = 0.65).

Polymorphisms at loci -31, -511, and -RN, and the morphology of coronary arteries.

The analysis of the connection between genetic variants at single loci and GS assessed at the first ACS did not show significant distribution differences—Table 7 and Table 8. Patients with three homozygous variants in the *IL-1RN* gene (11, 22 and 33) had the medians for GS 39.8, 38.5, and 49, respectively—Table 9. The analysis of genetic variants at the three loci showed the highest median for GS in the nine-patient group with the rare XS variant (-31CC/-511TT/-RN12)—80—and the lowest—38—in the group of patients with the L variant (-31CT/-511CT/-RN11). In the remaining frequent variants, the GS values were in the range 39.5–41.25—Table 10. 

The attempt to demonstrate the relationship of the number of coronary arteries showing ≥70% stenosis in coronary catheterization with the genetic variant has been negatively verified by the chi^2^ test—Table 7, Table 8 and Table 9. The analysis of the prevalence of collaterals depending on a single locus using Fisher’s exact test failed to disclose significant associations. Additionally, echocardiographic assessment of left ventricular hypertrophy did not confirm its relationship with the polymorphisms assessed in our study.

## 4. Discussion

The main finding of this study is a definition of the IL-1 gene cluster polymorphism profile, which in the cohort of Polish patients with ACS would be associated with a younger age of onset of the first ACS, as well as more advanced coronary artery atherosclerosis at the first coronary angiography performed in the course of ACS.

An important study on the number of alleles in terms of polymorphisms in the promotor region of the *IL-1B* gene is the publication which summarizes the results of genotyping from three major European programs designed to assess the environmental and genetic risk of MI [20]. Our result (34.2%) for the mutated T allele at locus -511 is almost identical to that obtained in the AIRGENE program—34.7%, where the researchers examined the genetic material of 392 inhabitants of Augsburg and Helsinki in 2003–2004. The distribution of alleles in the *IL-1RN* gene was compared with the data of Polish coronary patients and controls—inhabitants of West Pomerania Province [21], German patients with chronic type B gastritis (11% were immigrants from the Mediterranean region), and Finnish blood donors [22,23]. Size-wise, the groups were similar to ours (202 + 119, 210, and 338 people). The frequencies of allele 1 and of pair 11 were lower than those in our group, and yet comparable in the West Pomeranian and Finnish groups—65.9 and 67.4, i.e., 42.8% and 44.0%, respectively. Our results had the highest percentage of allele 1 and pair 11, and were the closest to those of the German gastric patients—the values were 77.4% and 73.6%, and 63.6% and 58.0%, respectively. Analogously, in our group and the German one, allele 2 and pair 22 were less frequent—18.6% and 22.1% and 6.8% and 8.1%, respectively—while in the groups reported by Gorący et al. and Eklund et al., they were 31.8% and 31.6% and 10.7% and 8.0%, respectively.

DNA changes at 50 loci of genes connected with proinflammatory phenomena were analyzed with respect to explaining the occurrence of premature coronary artery disease within the GRACE-IMMUNE program (Genetic Risk of Acute Coronary Events), which comprised 2699 patients [24]. That study tested a hypothesis on the influence of *IL-1B* polymorphisms on the appearance of ACS before 65, the result being weaker than for the classical risk factors, but still statistically significant. That study, however, focused on different polymorphisms than those examined in our study (loci +4336 and +1423). The influence of *IL-1B* polymorphisms at locus -511 on the risk of MI at a young age was studied in 400 Italian patients, where men had an MI before 45 and women before 50, and 134 patients with ischemic stroke before 45. It was demonstrated that the frequency of carriers of homozygous TT variant at locus -511 is significantly lower in the study group than in controls. That led the researchers to the conclusion that this genetic variant has a protective role against an early MI or stroke [25]. After some years, the genotyping of DNA samples of the same people was extended to include the analysis of other loci (two in the promotor region, two in the encoding unit of *IL-1B,* and one in the *IL-1RN* gene), which confirmed previous observations [26]. Although in our study, patients with this beneficial variant constituted only 9.6% of the study group, among them, we saw the lowest percentage of patients with an MI before 60 (Table 5). Each reduction of the number of T allele in a pair by 1 was associated with an approximately 4%-reduction of the percentage of such patients. This weak tendency, however, was not confirmed statistically. 

The *IL-1B* polymorphisms and results of coronary catheterization.

There are no available reports on the possible connection between *IL-1B* and *IL-1RN* polymorphisms and the accelerated progression of coronary atherosclerosis. Studying atherosclerotic changes in the coronary arteries of patients after an MI, the already-mentioned Olofsson et al. stated that a larger mean area of the atherosclerotic plaque was observed in carriers of pair 11 in the *IL-1RN* than in the carriers of the remaining variants (12 and 22) [27]. In our study, there were no analogous correlations.

Interesting results on the location of atherosclerosis and genetic variation in 9 SNP of such genes as TNF-alfa, IL-1b, IL-10, and TGF-b1 were obtained by pathomorphologists studying this phenomenon among Japanese patients. They observed a connection between a variant of the *IL-1B* at locus -511 and atherosclerosis only in the subclavian artery—OR 1,35 [CI95 1.01–1.8] *p* < 0.05, without confirming such a connection for changes in the coronary, intracerebral, carotid, femoral, internal, and external iliac arteries, in the aorta, the splenic artery, and the superior mesenteric artery. None of the polymorphisms examined had a significant effect on coronary atherosclerosis, whereas the significance of the classical risk factors (DM, HA, hyperlipidemia) was confirmed in 1500 post-mortems with the OR between 1.97–2.26 with *p* < 0.05 [28]. The importance of the C allele at the -31 locus for susceptibility to ischemic stroke was emphasized in a study by Gorący et al. [29].

The influence of *IL-1B* and *IL-1RN* polymorphisms on the presence of collaterals was tested in connection with the recent reports on in vitro basic research on the angiogenic properties of this cytokine [30,31]. The numerical differences of the percentages of patients with collaterals were not statistically significant. Although in a study by Ørn et al., nuclear magnetic resonance indicated a positive correlation between IL-1b concentrations and left ventricular mass index in myocardial infarction survivors, in our study, echocardiographic assessment of this parameter did not confirm its relationship with related polymorphisms of this cytokine [32,33].

### Study Limitations 

The main limitation of this study is the relatively small sample size due to the clinical focus in the selection of patients with a clinical diagnosis of the first ACS rather than a random population sample. One cannot argue with the result of the Genomewide Association Study, in which the SNP of the *IL-1B* failed as potential genetic markers for increased risk of IHD [34]. However, in the current study, we did not compare patients with ACS with controls without ACS, but we checked a selected demographic and angiographic feature. The literature on the subject showed that the influence of genetic variation on the specific parameters in MI survivors was significant, using a comparable sample size.

The next limitation of this study is an underrepresentation of women. The percentage of females is lower when compared with values reported in Polish ACS Registries [35,36]. Surprisingly, females were underrepresented in the five largest subgroups (XL, L, M, S, and XS) and overrepresented in the group of remaining individuals with variants of very low frequency. 

Our results could have been affected by the specific selection of patients—excluding patients with stable coronary artery disease who had never experienced an ACS and patients with severe conditions who died during the in-hospital treatment—so one cannot automatically extrapolate the results to the entire pool of patients with various forms of IHD. Information about the family history of coronary artery disease in first-degree relatives could enrich the interpretation of the results. These data were, however, incomplete among our patients—some of them were not able to define precisely the health problems in their families, or in some cases, nonmedical causes of death of their parents were declared. Additionally, the choice of Gensini Score for the quantification of coronary atherosclerosis diffuseness may be disputable, since other scores (such as the Bogaty score or Sullivan score) are also available, and they play a complementary role in these kinds of studies [37]. The Gensini Score was chosen, as these authors had used it in their previous studies on the impact of polymorphism of genes responsible for the severity of inflammatory reactions [38].

Another limitation might be the lack of reference to the actual IL-1b and IL-1RA concentrations in the studied patients, and the assumption that they are consistent with the in vitro measurements, described by the authors of earlier studies. One must be aware, however, that the clear formulation of the results and relating them to the patients’ clinical data required a simplification of certain dependencies, and that the concentrations of cytokines in the blood are very labile. 

In vitro, the IL-1b:IL-1RA concentration without lipopolysaccharide (LPS) stimulation is 1:100; after LPS stimulation, the IL-1b concentration increases approximately 20–30 times and, with a somewhat smaller increase in the IL-1RA concentration, the ratio is 1:80. The concentration of these cytokines is affected by the activities of daily living, stress, and especially the number of circulating monocytes. Thus, in order to obtain comparable results, one would have to establish strict conditions for the collection of blood samples for testing. Wen et al. have proved in vitro that when the LPS concentration does not affect monocytes, the basic IL-1b production is low and does not depend on the patient’s genotype [39]. However, concentrations of this cytokine were significantly different after the abovementioned bacterial stimulation in vitro. Studying human monocytes with the most frequent variants at loci -31, -511, and -1470, they obtained the following results: the highest intensification of IL-1b secretion was in wild homozygotes (-31TT/-511CC/-1470GG), the lowest in mutated homozygotes (-31CC/-511TT/-1470CC), and the mean intensification was in the monocytes of heterozygotic organisms. Subsequent studies have confirmed the correctness of these findings [40].

## 5. Conclusions

In terms of frequency of the rarer alleles in the *IL-1B* gene SNP at loci -31 and -511, and the *IL-1RN* VNTR polymorphism the group tested in our clinic did not differ from other ethnic groups described in the literature, with a single distinguishing feature of less frequent conjunction of variants of alleles at loci -31 and -511.

The nucleotide variation in the promotor region of the *IL-1B* gene at loci -31 and -511 and the VNTR polymorphism of its receptor antagonist gene can explain individual differences in the dynamics of IHD and differences in the progression of atherosclerosis of the coronary arteries undergoing invasive diagnostics because of ACS, especially for patients with a first ACS before 60.

## Figures and Tables

**Table 1:** The nomenclature used for the polymorphism of a variable number of tandem repeats.

Numeric Symbol	Number of 86 Base Pair Repeats
1	4
2	2
3	5
4	3
5	6

**Table 2:** Demographic data of the studied group.

Parameter/Variable	The Value/Frequency of Occurrence n (%)
Age (years) during the index ACS—range	39–83
The median age [interquartile range] (years)	59 [53–65]
Mean age ± SD (years)	59.2 ± 9
Males	187 (74.8%)

**Table 3:** Risk factors of atherosclerosis and other vascular or chronic inflammatory diseases in the history of the patients in the studied group.

Risk Factor/Disease Entity	Value/Frequency of Occurrence (%)
Arterial hypertension	180 (72.0%)
Type 2 diabetes mellitus	70 (28.0%)
Smoking	111 (44.4%)
Obesity	91 (36.4%)
BMI: minimum–maximum	18.4–41.1
BMI: median [interquartile range]	28.1 [25.5–31.0]
Hyperlipidemia	188 (75.2%)
Cerebral stroke	20 (8.0%)
Atherosclerosis of arteries of lower limbs	6 (2.4%)
Abdominal aortic aneurysm	6 (2.4%)
Intracranial aneurysm	9 (3.6%)
Chronic inflammatory diseases *	5 (2.0%)

* psoriasis, rheumatoid arthritis, ankylosing spondylitis.

**Table 5:** The observed distribution of the alleles and genotypes at loci -31 and -511 of the *IL-1B* and *IL-1RN* genes and the theoretical distribution of genotypes.

Locus	Allele/Genotype	The Real Number/Frequency of Alleles/Genotypes n (%)	The Theoretical Number/Frequency in Accordance with the Hardy-Weinberg Equation n (%)	Significance of the Differences in the Distribution of the Frequencies of Genotypes (chi^2^ Test)
-31	C/ T/ /CC /CT /TT	170 (34.0) 330 (66.0) 24 (9.6) 122 (48.8) 104 (41.6)	× × 28.9 (11.56) 112.2 (44.88) 108.9 (43.56)	*p* = 0.38
-511	T/ C/ /TT /CT /CC	171 (34.2) 329 (65.8) 24 (9.6) 123 (49.2) 103 (41.2)	× × 29.2 (11.7) 112.6 (45.0) 108.2 (43.3)	*p* = 0.34
-RN	1/ 2/ 3/ 4/ /11 /22 /33 /44 /12 /13 /14 /23 /24 /34	387 (77.4) 93 (18.6) 19 (3.8) 1 (0.2) 159 (63.6) 17 (6.8) 5 (2.0) 0 (0) 59 (23.6) 9 (3.6) 1 (0.4) 0 (0) 0 (0) 0 (0)	× × × × 149.7 (59.9) 8.6 (3.5) 0.4 (0.1) <0.1 (<0.1) 71.9 (28.8) 14.7 (5.8) 0.8 (0.3) 3.5 (1.4) 0.2 (<0.1) < 0.1 (<0.1)	*p* = 0.0001

**Table 6:** The observed frequency of occurrence of the variants of three polymorphisms with the frequency resulting from the Hardy-Weinberg equation with the assessment of the significance of differences.

No.	The Variant with Three Polymorphisms -31 -511 -RN	The Real Number and Frequency n (%) Letter Symbol	The Theoretical Number and Frequency in Accordance with the Hardy-Weinberg Equation n (%)	The Significance of Distribution Differences of the Genetic Variants—Real and Theoretical (chi^2^ Test)
	69 Variants not Represented in the Study Group	0 (0)	72.62 (29.06)	
i ii iii iv v vi vii viii ix x xi xii xiii xiv xv xvi xvi xviii xix xx xxi	CT CT 11 TT CT 11 CT CC 11 TT CC 11 CT CT 12 TT CC 12 TT CC 13 CT TT 11 CC CT 11 CC CT 12 CT CT 13 CC TT 11 CT CT 22 TT CC 22 CC TT 12 CT TT 22 TT CC 14 CC TT 22 CT CT 33 CT TT 33 TT CC 33	67 (26.8) L 3 (1.2) 2 (0.8) 78 (31.2) XL 39 (15.6) M 10 (4.0) S 6 (2.4) 1 (0.4) 2 (0.8) 1 (0.4) 3 (1.2) 6 (2.4) 5 (2.0) 5 (2.0) 9 (3.6) XS 1 (0.4) 1 (0.4) 6 (2.4) 3 (1.2) 1 (0.4) 1 (0.4)	30.25 (12.1) 29.36 (11.7) 29.1 (11.6) 28.24 (11.3) 14.53 (5.8) 13.57 (5.4) 2.77 (1.1) 7.86 (3.1) 7.79 (3.1) 3.74 (1.5) 2.97 (1.2) 2.02 (0.8) 1.74 (0.7) 1.63 (0.6) 0.97 (0.4) 0.45 (0.2) 0.14 (<0.1) 0.12 (<0.1) 0.07 (<0.1) 0.01 (<0.1) 0.01 (<0.1)	<0.001

**Table 7:** The clinical, angiographic, and echocardiographic parameters according to the variant at locus -31.

The Parameter		-31 TT n = 104	-31 CT n = 122	-31 CC n = 24	Significance (chi^2^ Test)
ASC <60 years of age	n	57	60	12	0.69
	%	54.8	49.2	50	
Collateral circulation	n	35	45	10	0.67
	%	34.0	37.2	43.5	
Gensini Score	mean ± SD	48 ± 35	45 ± 29	51 ± 41	0.94
	Minimum–maximum	1–188	4–148	6–140	
	The median [25–75%]	40 [24–65]	39 [24–63]	34 [19–83]	
The number of arteries with stenosis ≥70%: 0/1/2/3	The number of patients	4/39/38/23	1/61/40/20	0/14/5/5	0.76
	%	4/37.5/36.5/22	0.8/50/32.8/16.4	0/58/21/21	
Left ventricular hypertrophy: absent/present	n	15/110	12/87	6/20	0.81 ^1^
	%	6/44	4.8/34.8	2.4/8	

^1^ Fisher’s exact test.

**Table 8:** The clinical, angiographic, and echocardiographic parameters according to the variant at locus -511.

The Parameter		-511 CC n = 103	-511 CT n = 123	-511 TT n = 24	Significance (chi^2^ Test)
ASC <60 years of age	n	56	62	11	0.70
	%	54.4	50.4	45.8	
Collateral circulation	n	34	46	10	0.66
	%	33.3	38.0	41.6	
Gensini Score	mean ± SD	47 ± 34	47 ± 31	51 ± 41	0.99
	Minimum–maximum	1–188	4–154	6–140	
	The median [25–75%]	40 [23–62]	39 [24–66]	37 [20–80]	
The number of arteries with stenosis ≥70%: 0/1/2/3	The number of patients	4/40/36/23	1/61/41/20	0/13/6/5	0.88
	%	3.9/38.8/35/22.3	0.8/49.6/33.3/16.3	0/54/25/21	
Left ventricular hypertrophy: absent/present	n	15/110	12/87	6/20	0.81 ^1^
	%	6/44	4.8/34.8	2.4/8	

^1^ Fisher’s exact test.

**Table 9:** The clinical and angiographic parameters according to the variant at locus -RN.

The Parameter		RN 11 n = 159	RN 12 n = 59	RN 22 n = 17	RN 13 n = 9	RN 33 n = 5	RN 14 n = 1	Significance (chi^2^ Test)
ASC < 60 years of age	N	77	40	5	5	2	0	0.04
	%	48.4	67.8	29.4	55.6	40.0	0	
Collateral circulation	N	56	21	7	4	2	0	0.94
	%	35.7	35.6	43.7	44.4	40	0	
Gensini Score	mean ± SD	48.4 ± 34.0	48.2 ± 34.3	39.4 ± 22.3	42.1 ± 33.8	48.2 ± 19.3	14	0.78
	Minimum–maximum	3.5–188	1–148	6–80	2–88	24–77	14–14	
	The median [25–75%]	39.8 [24–74]	40.0 [24–80]	38.5 [23–54]	36.5 [4–80]	49 [40–51]	14	
The number of arteries with stenosis ≥70%: 0/1/2/3	The number of patients	2/68/56/33	1/32/15/11	0/9/6/2	2/2/4/1	0/2/2/1	0/0/1/0	0.99
	%	1.2/42.8/ /35.3/ /20.7	1.6/54.3/ /25.5/ /18.6	0/53/35/ /12	22.2/22.2/ /44.5/11.1	0/40/40/20	0/0/100//0	
Left ventricular hypertrophy: absent/present	N	29/130	12/47	6/11	2/7	1/4	0/1	0.87 ^1^
	%	11.6/52	4.8/18.8	2.4/4.4	0.8/2.8	0.4/1.6	0/0.4	

^1^ Fisher’s exact test.

**Table 10:** The clinical and angiographic parameters according to the variants at the three loci—the significance was calculated using Fisher’s exact test for 250 people, but the table contains data about the five most numerous variants.

The Parameter		The Genetic Variant at the 3 Loci Numbers in the Subgroups Variant Symbol					Significance (Fisher’s Exact Test)
		-31 TT -511 CC -RN 11 n = 78 XL	-31 CT -511 CT -RN 11 n = 67 L	-31 CT -511 CT -RN 12 n = 39 M	-31 TT -511 CC -RN 12 n = 10 S	-31 CC -511 TT -RN 12 n = 9 XS	
ACS <60 years of age	n	46	25	28	6	5	0.07
	%	58.9	37.3	71.8	60	55.6	
Collateral circulation	N	26	24	14	3	4	0.63
	%	33.3	36.4	35.9	30.0	44.4	
Gensini Score	mean ± SD	50.2 ± 35.8	43.8 ± 28.1	46.9 ± 34.1	36.9 ± 18.4	69.6 ± 43.2	0.62
	Minimum–maximum	3.5–188	4–112	4–148	1–59	16–140	
	The median [25–75%]	40 [24–80]	38 [24–64]	39.5 [24–80]	41.25 [21–52]	80 [32–86]	0.58
	% of distribution acc. to the tertiles 1:2:3	33:31:36	34:32:34	36:32:32	34:30:36	22:22:56	
The number of arteries with stenosis >70%: 0/1/2/3	Number of patients	1/31/27/ /19	1/29/26/ /11	0/25/9/5	1/5/2/2	0/2/3/4	0.97
	%	1.4/39.7/ /34.6/24.3	1.6/43.2// /38.8/16.4	0/64.1/ /23.1/12.8	10/50/ /20/20	0/22.2/ /33.3/44.5	

**Table 4:** Data related to the diagnosis and treatment of the acute coronary syndrome.

Parameter/Result of Examination/Treatment	The Value/Frequency of Occurrence n (%)
ACS ^1^ with ST segment elevation	177 (70.8%)
ACS without ST segment elevation	73 (29.2%)
Primary PCI ^2^ with stent implantation	193 (77.2%)
Primary balloon PCI	4 (1.6%)
Pharmacological treatment of ACS alone	19 (7.6%)
Surgical revascularization after ACS (in three patients after pPCI ^2^)	37 (14.8%)
Gensini Score Minimum–maximum median [interquartile range] mean ± SD	1–188 40.0 [24.0–66.0] 47.45 ± 33.1
Left ventricular ejection fraction (%) Minimum–maximum median [interquartile range] mean ± SD	22–60 47 [40–54] 46.5 ± 9.5
Left ventricular hypertrophy	217 (86.8%)

^1^ ACS—acute coronary syndrome; ^2^ PCI—percutaneous coronary intervention.

## Data Availability

The data presented in this study are available on request from the corresponding author. The data are not publicly available due to privacy restrictions.
